# Multi-classification of national fitness test grades based on statistical analysis and machine learning

**DOI:** 10.1371/journal.pone.0295674

**Published:** 2023-12-22

**Authors:** Qian Yang, Xueli Wang, Xianbing Cao, Shuai Liu, Feng Xie, Yumei Li

**Affiliations:** School of Mathematics and Statistics, Beijing Technology and Business University, Beijing, China; New York University Abu Dhabi, MALAYSIA

## Abstract

Physical fitness is a key element of a healthy life, and being overweight or lacking physical exercise will lead to health problems. Therefore, assessing an individual’s physical health status from a non-medical, cost-effective perspective is essential. This paper aimed to evaluate the national physical health status through national physical examination data, selecting 12 indicators to divide the physical health status into four levels: excellent, good, pass, and fail. The existing challenge lies in the fact that most literature on physical fitness assessment mainly focuses on the two major groups of sports athletes and school students. Unfortunately, there is no reasonable index system has been constructed. The evaluation method has limitations and cannot be applied to other groups. This paper builds a reasonable health indicator system based on national physical examination data, breaks group restrictions, studies national groups, and hopes to use machine learning models to provide helpful health suggestions for citizens to measure their physical status. We analyzed the significance of the selected indicators through nonparametric tests and exploratory statistical analysis. We used seven machine learning models to obtain the best multi-classification model for the physical fitness test level. Comprehensive research showed that MLP has the best classification effect, with macro-precision reaching 74.4% and micro-precision reaching 72.8%. Furthermore, the recall rates are also above 70%, and the Hamming loss is the smallest, i.e., 0.272. The practical implications of these findings are significant. Individuals can use the classification model to understand their physical fitness level and status, exercise appropriately according to the measurement indicators, and adjust their lifestyle, which is an important aspect of health management.

## Introduction

Physical fitness is a key element of a healthy life, and being overweight or lacking physical exercise will lead to health problems, so assessing physical fitness based on machine learning and data mining methods will have good application results. According to the National Physique Monitoring Bulletin, published for the fifth time in 2020 [1], the overall national physical fitness level is improving, however, the excessive growth rate of overweight and obesity is the primary problem of the national physical quality [2, 3]. There is extensive literature demonstrating the health benefits of participating in sports, but each sport should assess the physical fitness of the participants [4, 5]. If one possesses muscular physical fitness, it can enhance one’s athletic performance and help maintain a healthier physical condition.

The implementation of a national physical fitness test can help evaluate physical conditions and effects of exercise, scientifically guiding the population to engage in sports activities and enhance their fitness. Gao et al. used a self-organizing characteristic map (SOM) network to classify students’ physical level test scores and concluded that weight was the main factor affecting physical health [6]. Physical health is one of the critical elements of a healthy life, Sulla-Torres et al. used a neural network and fuzzy logic to classify the physical health status of 1813 children [7]. Sun et al. used a physique classifier based on a naive Bayes algorithm, divided students’ physical health status into four levels, concluded that the accuracy rate of the naive Bayes classification model reached 81.02%, and made suggestions for the development of school physical education, facilitating the timely detection of health problems [8]. Some recent research has used supervised machine learning and neural networks for health classification. Staudenmayer et al. used an improved artificial neural network to divide physical activities completed by subjects within a specified time into four activity types, with a classification accuracy of over 80% [9]. Saez et al. used the physical activity detection data of the aging population to construct a classification model for physical activity using k-nearest neighbor, naive Bayes, linear discriminant analysis, support vector machine, random forest, capacity enhancement tree, stochastic gradient descent, and neural networks [10]. Cai et al. obtained the overall health-level data of 890 adults through questionnaires and used machine learning methods such as logistic regression, deep learning, random forest, and gradient enhancement tree to establish a classification model, with accuracy higher than 85% [11].

Classification using machine learning techniques has been applied in various fields. It is efficient and accurate, and it enables model visualization. AlDahoul N et al. used various machine learning models to classify streamflow [12]. Khan et al. used the support vector machine, naive Bayes, multilayer perceptron, and decision tree to classify students’ early grades, and found that the decision tree classifier had high accuracy [13]. Hou et al. used four machine learning methods—gradient lifting model, random forest, deep neural network, and logistic regression—to classify the datasets of breast cancer cases and match healthy control groups [14]. Liu et al. utilized three public datasets containing 12 subsets to train multiclass emotion classifiers using five machine learning algorithms: support vector machine, neural network, naive Bayes, decision tree, and k-nearest neighbor [15]. Chicco et al. predicted the diagnosis of hepatitis C patients by analyzing their electronic medical records and utilizing three machine learning classifiers: random forest, logistic regression, and decision tree [16]. Singh et al. used seven classifiers—k-nearest neighbor, support vector machine, decision tree, naive Bayes, random gradient descent, random forest, and multilayer perceptron—to classify lung cancer image datasets, and the results showed that the accuracy of the multilayer perceptron classifier was highest [17]. Raza A et al. apply machine learning models to network attacks and propose a new classifier [18, 19]. Through the above comprehensive literature analysis, we found the following research gaps: (1) Although there is existing literature on physical health, their focus is mainly on students and professional athletes, and their research methods cannot be applied to other groups. No indicator system affects physical fitness test results, nor is there any order of importance for indicators that affect physical health. (2) Classification work using machine learning has been widely used in various fields, and the accuracy of the classification model is very high. However, there are currently few studies on applying machine learning classification models to national physical examination and health. In the 21st century, health issues are becoming increasingly important; national health is actual and meaningful. This paper used statistical analysis and machine learning methods to conduct multi-classification of national physical fitness test results without group restrictions, providing a scientific basis for exercise personnel to carry out sports. The work in this paper has the following advantages:

Statistical analysis and machine learning methods were used for the first time to classify national physical fitness test results. As long as physical fitness test data is available, physical health status can be assessed, providing a scientific basis for exercise personnel to carry out sports.

The selected measurement indicators come from a social physical fitness test survey to measure national health, and their indicators are scientific and practical. Through nonparametric tests, exploratory statistical analysis and feature importance scores, the measurement indicators with a significant impact on the grade of physical fitness test can be obtained to construct a reasonable index indicators.

Among many widely accepted and easy-to-implement machine learning algorithms, the best classification model can be selected to classify the national fitness test result grade and rank the importance of measurement indicators. Individuals can understand their physical fitness grade and status using a classification model in the early stage.

Therefore, methods based on statistical analysis and machine learning will have sound application effects in assessing physical conditions. The structure of this paper is as follows: The section “Materials and methods” mainly introduces the processing of data sets, multi-classification models, and evaluation indicators; Section “Results and discussion” compares and discusses the results of the applied methods and draws our main findings; Section “Conclusion” summarises the research conclusions limitations and future work.

## Materials and methods

**Fig 1:**
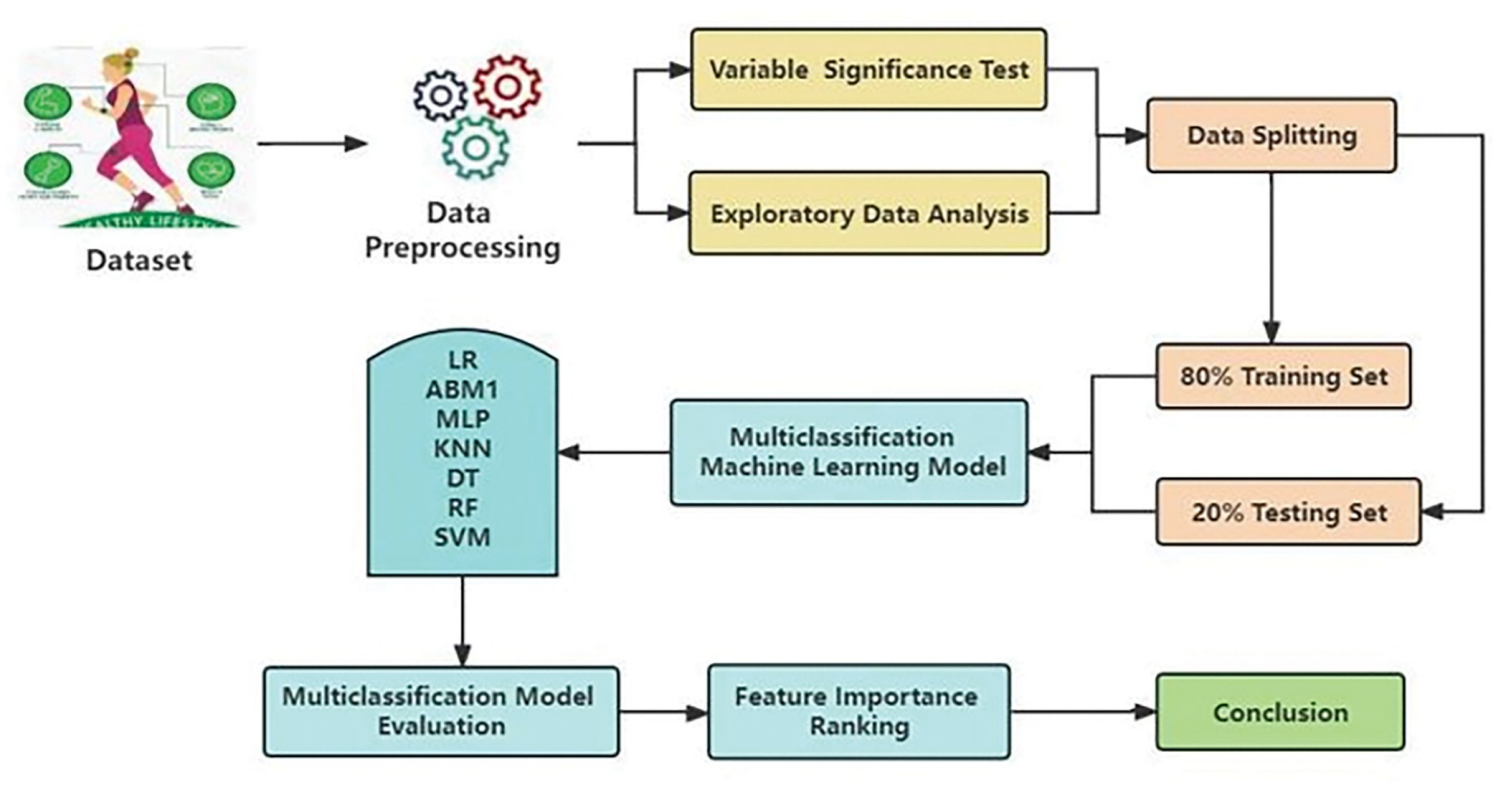
Flowchart of physical fitness test result classification.

This section covers the methodology of the presented work, as illustrated in a flowchart in Fig 1. Firstly, we preprocessed the national physical test data and then conducted parameter significance testing and exploratory statistical analysis. Secondly, we introduced the construction of multi-classification machine models and the tuning of hyperparameters; 80% of the data was used for training models, and 20% of the data was used for evaluating models. Finally, there was the model evaluation and the ranking of the importance of physical fitness test indicators.

### Data

#### Data collection

In this study, physique data measuring national health were used to build a classification model. The data come from the Kaggle website and contain 13,393 samples. Test results are divided into four grades, A through D, each accounting for 25% of the people, in descending order of score. The data include 12 features, ten quantitative and two qualitative, as described in Table 1.

**Table 1:** Variable descriptions.

Variable	Type	Description
Age	Continuous	Age at time of test
Gender	Categorical	Male = 1; Female = 0
Weight	Continuous	Body weight at test (kg)
Height	Continuous	Body height at test (cm)
Body fat	Continuous	Ratio of fat content to body weight
Diastolic	Continuous	Blood pressure during test
Systolic	Continuous	Lateral pressure of blood to blood vessel wall
Grip force	Continuous	Maximum grip value measured by squeeze hand force meter
Sit-bend forward	Continuous	Flexibility of human body (cm)
Sit-ups	Discrete	Number of sit-ups in 2 minutes
Broad jump	Continuous	No running long jump from a standing position (cm)
Target (class)	Categorical	A = excellent; B = good; C = pass; D = fail

#### Data preprocessing

Data preprocessing is an essential step for building a model; we perform missing value processing and outlier detection on sample data. Since no missing values in the data, we use a box diagram for outlier detection [20].

$$IQR=Q_{3}-Q_{1}$$ (1)

$$MI=Q_{1}-1.5*IQR$$ (2)

$$MA=Q_{3}+1.5*IQR$$ (3)

**Fig 2:**
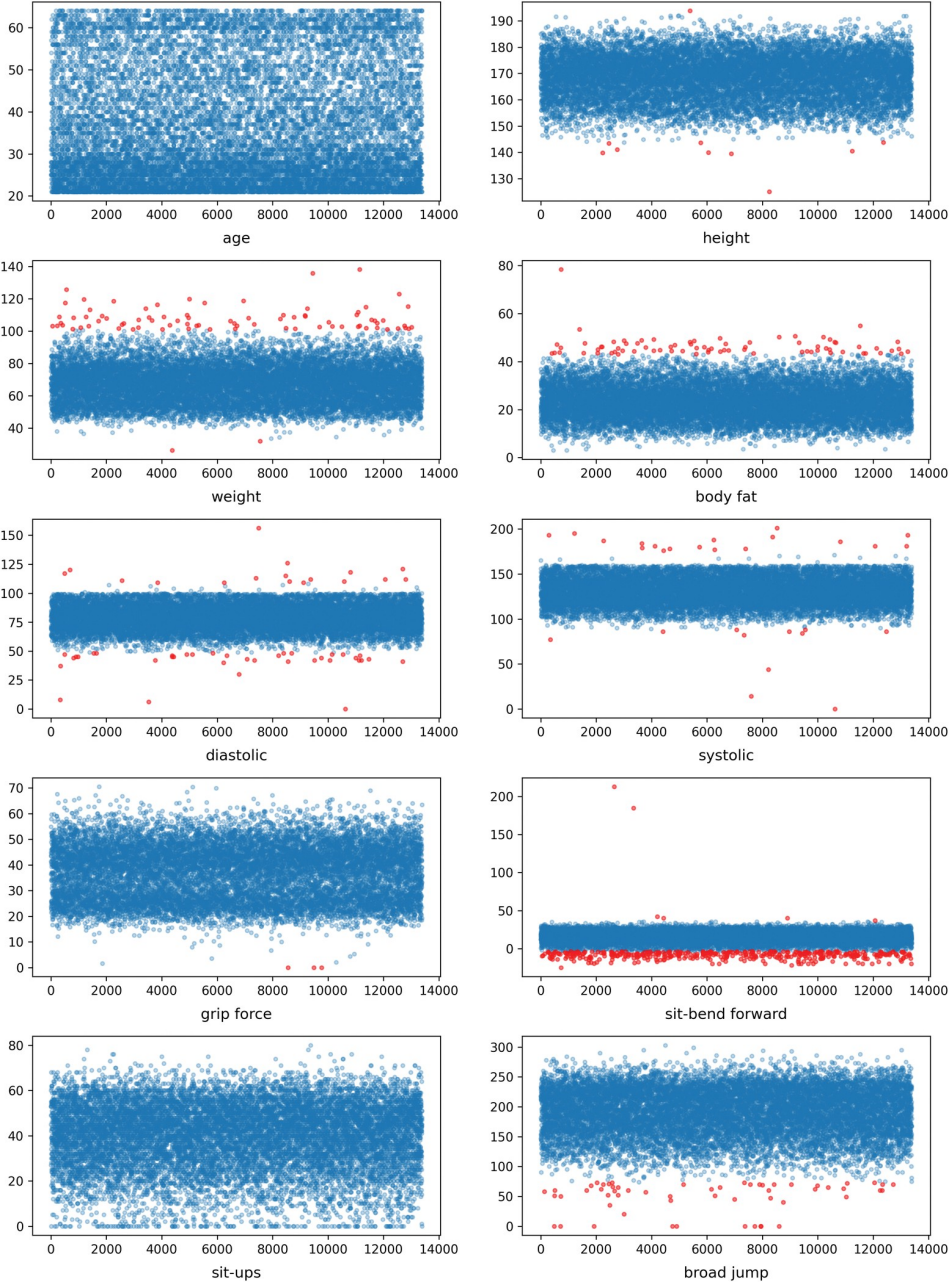
Abnormal value detection of physical fitness test indicators. Red points: abnormal; blue points: normal.

From the box diagram, we can roughly see the degree of dispersion of the data distribution. According to the upper and lower quartiles, we can obtain the interquartile range (IQR) and then calculate the lower limit (MI) and upper limit (MA), where values less than MI or greater than MA were outliers. The data distribution is shown in Fig 2, where red points are abnormal values, blue points are normal values, a red dot above a blue dot is an abnormally high value, and a red dot below a blue dot is an abnormally low value. We found that no abnormal value existed in the age and sit-ups, while the most abnormal value was sit-bend forward, followed by the weight and body fat variables.

After deleting outliers, we obtained 12,524 final samples, and the numbers of people with physical fitness test grades A, B, C, and D were 3316, 3313, 3318, and 2577, respectively. We conducted a statistical analysis and drew a heat map to reflect the correlation between data characteristics.

### Analysis of physical fitness test data

#### Data feature analysis

To understand the data characteristic distribution of the four groups, we use IQR to describe the distribution level of continuous and discrete variables, and we used percentages to describe the distribution level of classified variables. To compare the characteristics of the four groups, we used the Kruskal-Wallis (K-W) test for continuous and discrete variables and a chi-square test for classified variables. The K-W test is used as a substitute for the analysis of the variance of multiple independent samples when normal distribution conditions have not been met [21]. The chi-square test can identify significant differences in categorical variables between two or more groups [22]. All the test results are shown in Table 2, from which we can see that with the increase in weight and body fat, the physical fitness test grade gradually moved from excellent to fail. For the number of sit-ups and sit-bend forward, the better the fitness test grade, the higher the value. At the significance level of 0.05, all variables passed the significance test, indicating that the distributions of these variables (i.e., age, gender, weight, height, body fat, diastolic, systolic, grip force, sit-bend forward, sit-ups, and broad jump) across the four physical test levels were significantly different.

**Table 2:** Characteristics of groups A–D. At a 0.05 significance level, the K-W test was used for continuous and discrete variables, and the chi-square test was used for classified variables.

Variable	Group A	Group B	Group C	Group D	P value
Age, median (IQR)	30 (25–43)	32 (25–48)	32 (24–48)	35 (25–49)	<0.001
Gender n (%)	---	---	---	---	<0.000
Male	1842 (56%)	2143 (65%)	2221 (67%)	1709 (66%)	---
Female	1474 (44%)	1170 (35%)	1097 (33%)	868 (34%)	---
Weight (kg), median (IQR)	56 (51–64)	58 (52–67)	58 (52–67)	71 (62–80)	<0.001
Height (cm), median (IQR)	168 (162–174)	169 (163–174)	170(163–175)	170(162–175)	<0.001
Body fat (%), median (IQR)	20 (16–25)	22 (17–27)	22 (18–26)	27 (23–32)	<0.001
Diastolic (mmHg), median (IQR)	78 (71–86)	79 (71–86)	79 (71–86)	80 (73–88)	<0.001
Systolic (mmHg), median (IQR)	128 (119–140)	130 (120–141)	130(120–140)	131(120–143)	<0.001
Grip force, median (IQR)	39 (29–48)	40 (28–46)	38 (27–44)	36 (26–43)	<0.001
Sit-bend forward (cm),median(IQR)	21 (18–24)	17 (14–21)	14 (10–19)	10 (4–15)	<0.001
Sit-ups (number), median (IQR)	49 (40–56)	44 (34–51)	40 (30–48)	31 (21–42)	<0.001
Broad jump (cm), median (IQR)	202 (174–234)	200 (167–224)	194(160–220)	182(149–208)	<0.001

#### Exploratory data analysis

Exploratory data analysis(EDA) refers to the estimation and description of the relationship between a data distribution pattern, digital characteristics, and random variables through a graph, which facilitates the understanding of data characteristics.

**Fig 3:**
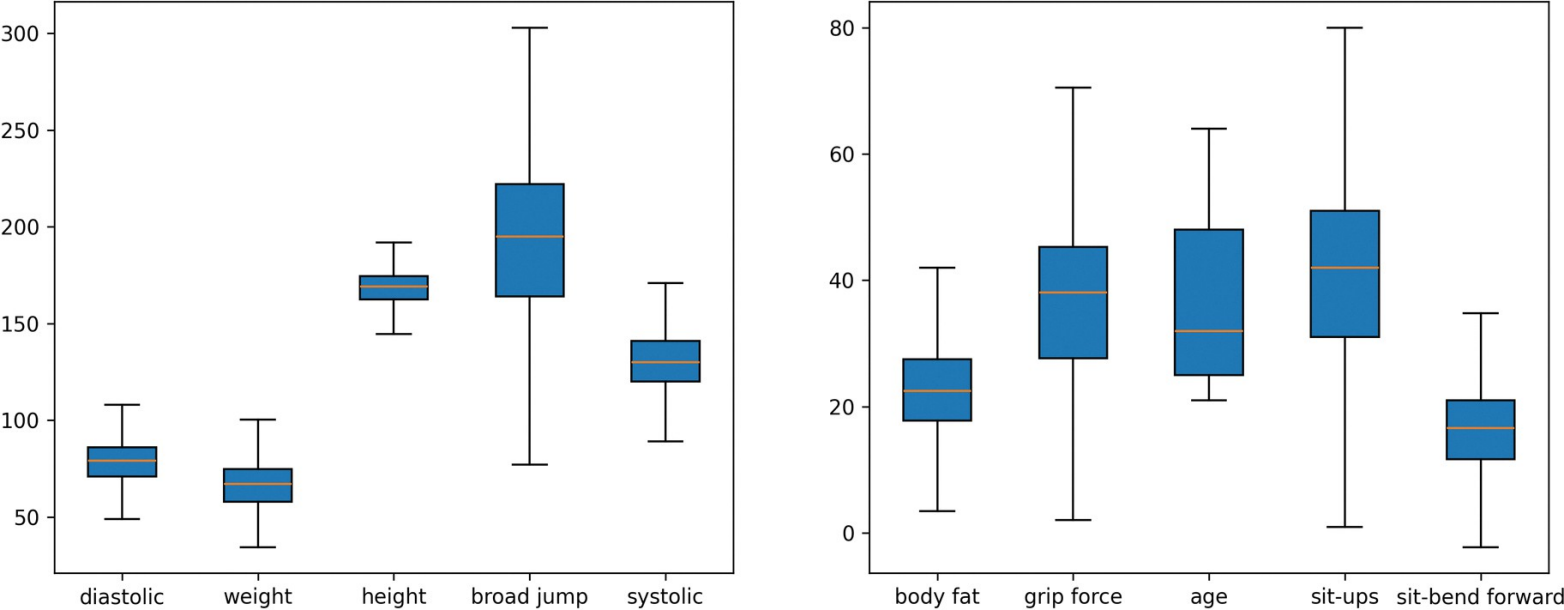
Box diagram after removing outliers.

Fig 3 shows the box plot distribution of ten quantitative variables after deleting outliers. The width of the box reflects the fluctuation of the data; the longer the box, the stronger the fluctuation. The variable with the largest fluctuation was the broad jump.

**Fig 4:**
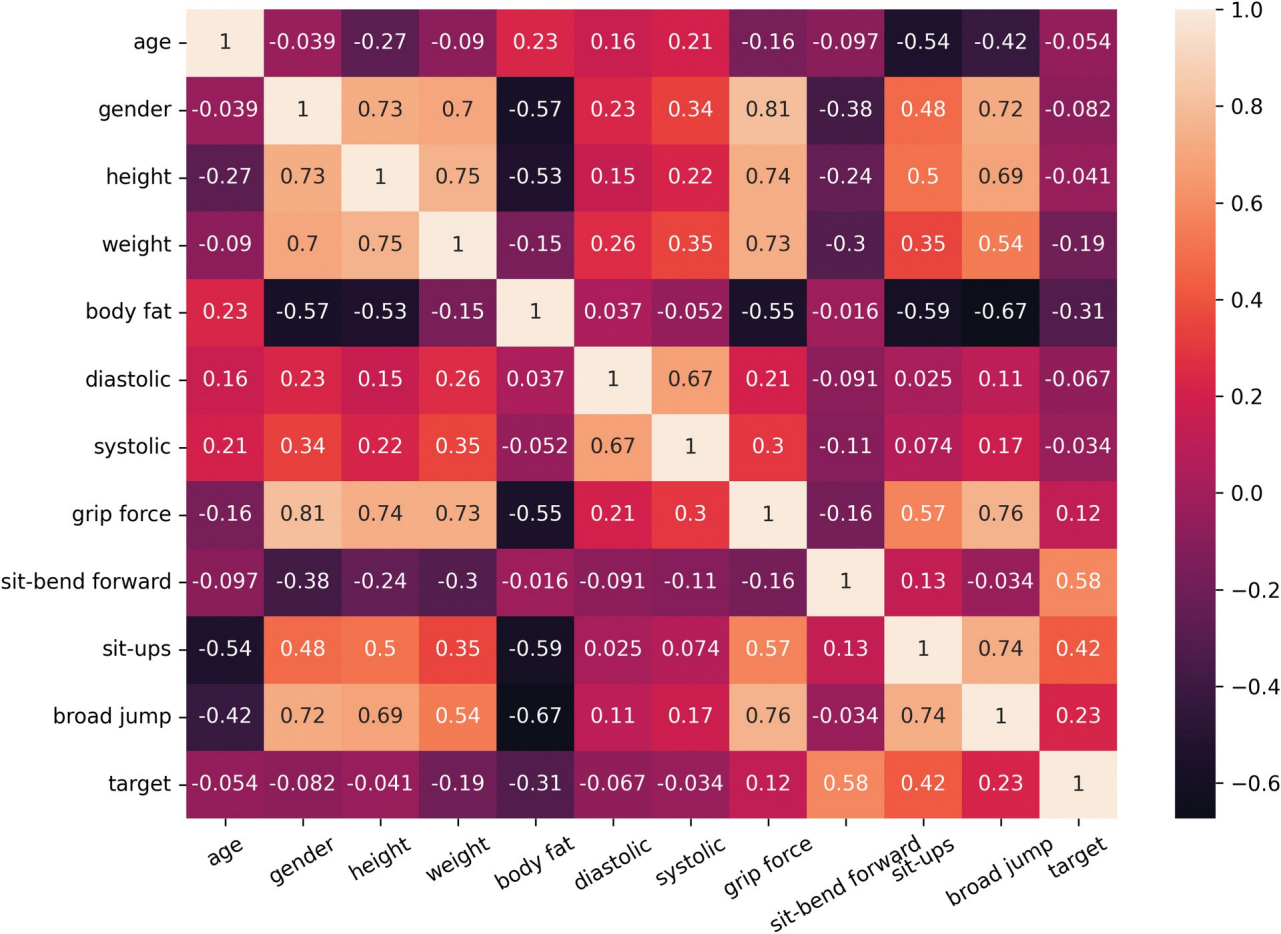
Sample correlation heat map.

Correlation is analyzed through the display of the numerical matrix of two variables through the heat map. The darker the color, the stronger the negative correlation, and the lighter the color, the stronger the positive correlation. It can be seen from Fig 4 that the number of sit-bend forwards was positively correlated with the classification of physical fitness, and that weight and body fat were negatively correlated with it.

**Fig 5:**
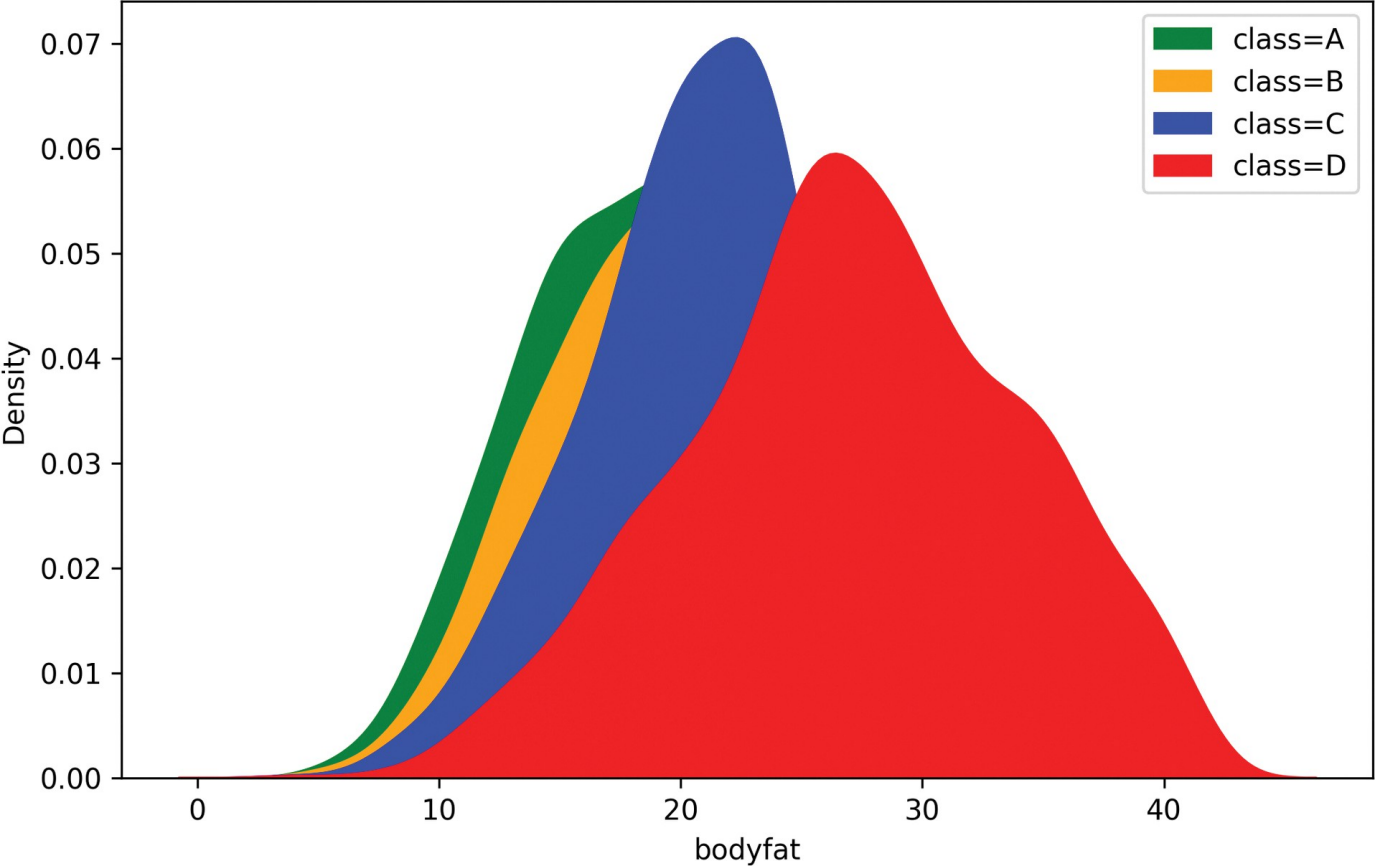
Body fat ratio density distribution map.

Fig 5 shows the density distribution of body fat in datasets under different categories of physical fitness test results. From Fig 5, it can be seen that the higher the body fat, the worse the grade in the physical fitness test results. When the body fat was higher than 25, the number of people in the worst grade of the physical fitness test was the largest. Therefore, body fat is an important factor in evaluating the physical fitness level and is closely related to health status and sports performance.

**Fig 6:**
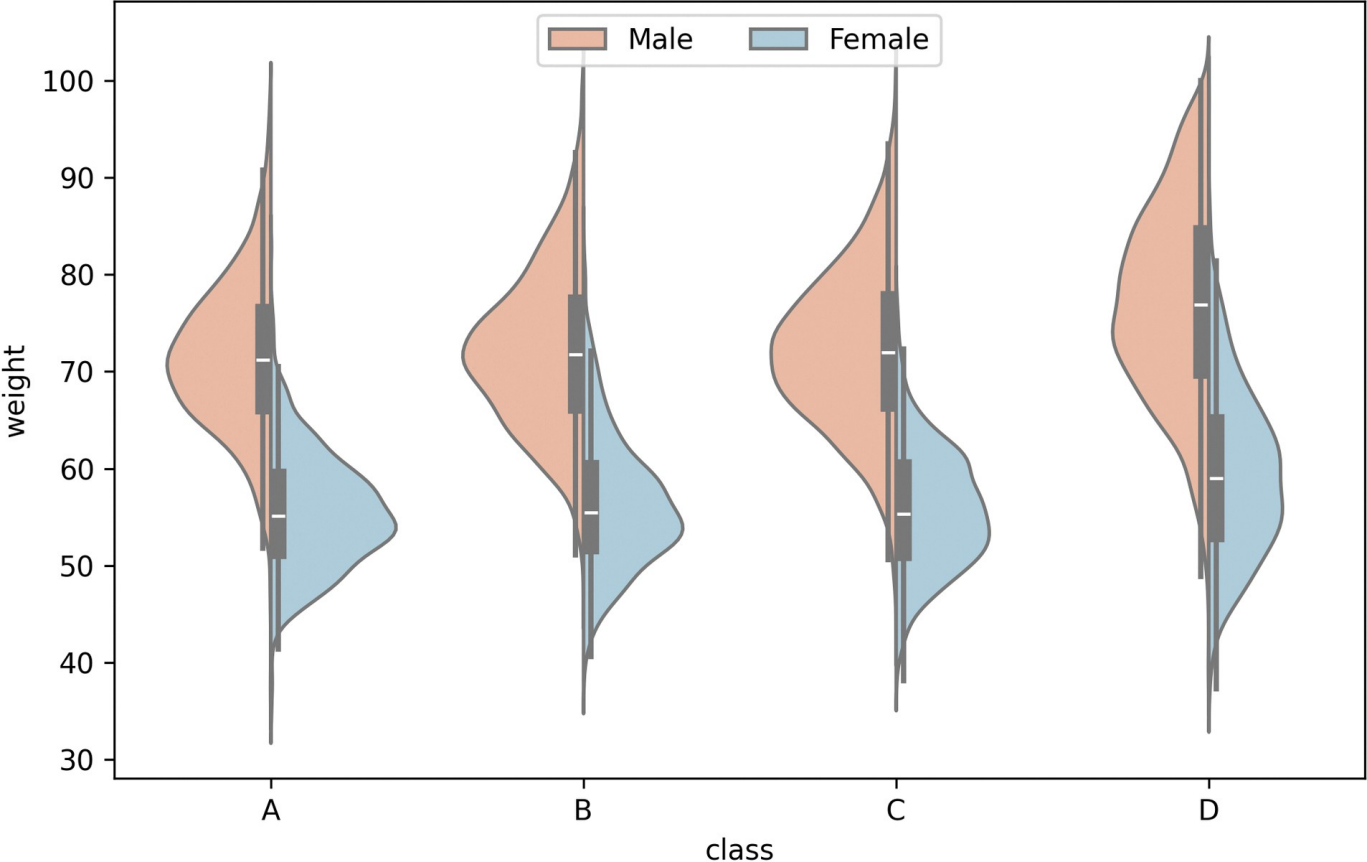
Constitution distribution map under different genders.

Weight management is also an important aspect of contemporary health management. Fig 6 shows the weight distribution corresponding to the four test levels. Whether male or female, if the median weight of each category represents the overall weight level of the group, when the physical fitness test results were grade A, the weight of the group was the least. When the physical fitness test results were the worst, i.e., grade D, the weight value was the highest and the data distribution is relatively discrete. Therefore, it can be concluded that weight is negatively correlated with physical fitness test results, and that proper control of weight will have a positive effect on the physical fitness level.

### Model construction

#### Multi-classification model

**Fig 7:**
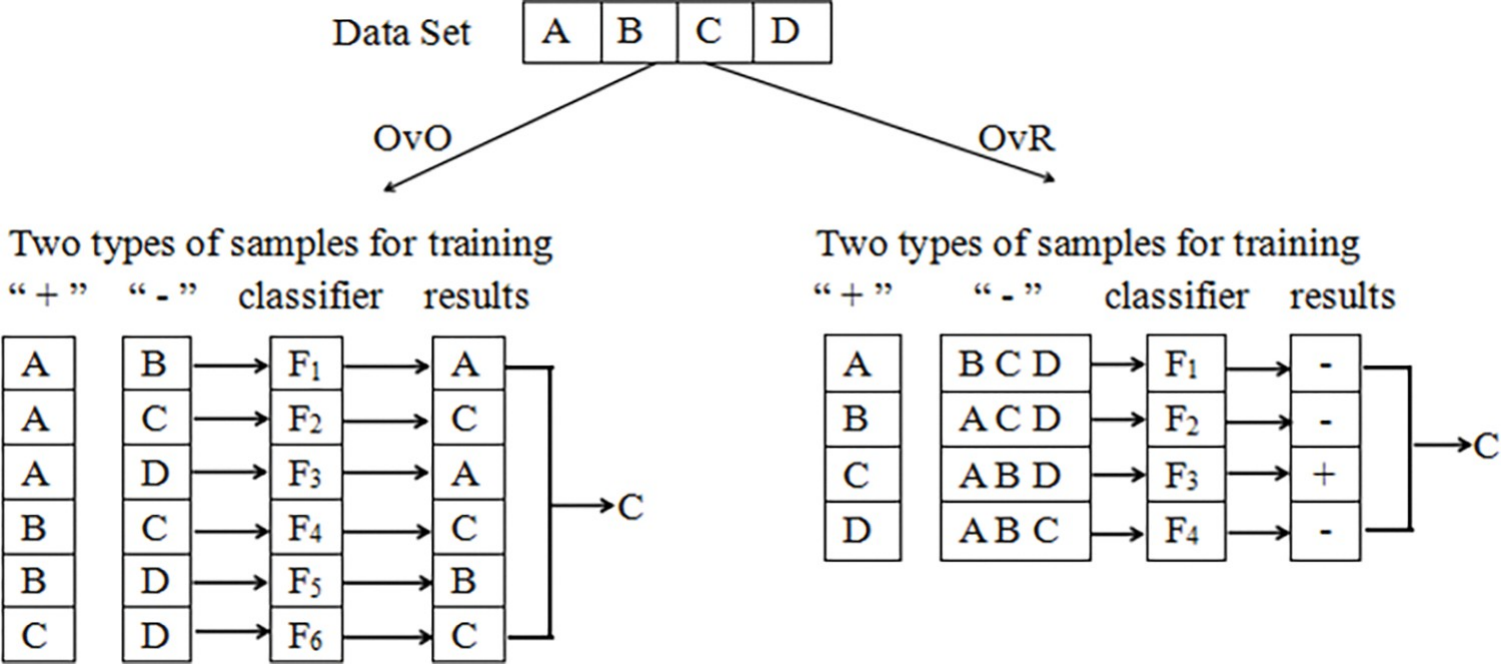
Multi-classification strategy map.

Multi-classification problems distinguish more than two category labels and are most commonly solved by transforming them into binary classification problems, using either a one-vs-the-rest or one-vs-one approach. The classification strategy is shown in Fig 7. We use the one-vs-the-rest method, where one of n categories is selected, and the other n − 1 are taken as a category. This is done n times. One-vs-one means selecting any two of n categories for classification, using the binary classification method to judge which of the two categories the sample belongs to, and recording the classification results. $C_{n}^{2}$ times are required to record all the classification results, and the result with the largest proportion is the final result.

In this paper, seven classic machine learning algorithms were used to build a multi-classification model: logistic regression (LR), adaboostm1 (ABM1), multilayer perceptron (MLP), k-nearest neighbor (KNN), decision tree (DT), random forest (RF), and support vector machine (SVM). Grid search was used to select the optimal super-parameter combination of the model, and ten fold cross-validation was performed. The super-parameter combination with the highest average score was taken as the best choice, and the best super-parameter combination was substituted in the model, which was used to train and test the datasets. The optimal hyperparameter settings of the seven machine learning models are shown in Table 3.

1. Logistic regression
  Logistic regression is a robust classifier in supervised machine learning algorithms. The main idea is to establish a regression formula for the classification boundary based on the existing data to perform classification. Not only the classification interval is given, but also the posterior probability of the class to which the sample belongs [23].
2. AdaboostM1
  The AdaboostM1 algorithm is an improved version of the Adaboost algorithm. It is a supervised machine learning classifier based on ensemble learning. All generated weak classifiers are combined using a linear combination method to form a robust classifier [24].
3. Multilayer perceptron
  A simple perceptron can be regarded as a two-class classification problem. If the output is multi-class, it will become a softmax regression. If a hidden layer is added to the softmax regression, it will become the multilayer perceptron used in this paper. The number of layers of multilayer perceptron and the hidden units in each hidden layer were super-parameters [25]. In this paper, the default super-parameter combination was used for sample classification.
4. K-nearest neighbor
  The k-nearest neighbor algorithm is a generalized algorithm for the nearest neighbor rule. In the decision-making stage, the nearest neighbor is extended to k, which is generally measured by the Euclidean distance formula [26]. If most of the k sample instances belong to a specific category, the sample instance is input classified under this category [27].
5. Decision tree
  Decision tree is a hierarchical form of instances and attributes that can be used for classification and prediction problems. Each decision node is a prediction value or classification value [28]. The classification algorithm divides the samples into appropriate sub-nodes, where the testing and branching process is repeated until it reaches the leaf node. The leaf node or terminal node will correspond to the decision result, which is the final classification result [29].
6. Random forest
  Random forest can be summarized as a classifier based on ensemble learning, which uses a number of decision trees on different subsets to find the best features with high accuracy and prevent over-fitting problems [30].
7. Support vector machine
  Support vector machine can be regarded as a linear classifier with maximum margin for classification problems. It is based on the concept of a decision plane that represents the decision boundary for successfully handling a classification problem [28, 31].

**Table 3:** Hyperparameter settings for seven machine learning models.

Machine learning model	Hyperparameter value
LR	C = 1, multi_class = ’ovr’
ABM1	learning_rate = 1, n_estimators = 200
MLP	default value
KNN	leaf_size = 20, n_neighbors = 10, p = 1, weights = ’distance’
DT	max_depth = 10, min_samples_leaf = 7, min_samples_split = 5
RF	max_depth = 100, min_samples_leaf = 1, min_samples_split = 2, n_estimators = 100
SVM	decision_function_shape = ’ovr’, C = 10, kernel = ’linear’

#### Performance evaluation metric

To obtain the best classification model, we built models using the seven machine learning algorithms on the training set, and we used the test set to derive some evaluation indicators. Recall is the proportion of correctly identified positive samples [32]. Micro-recall refers to adding the number of correctly classified samples in each binary model and dividing it by the total number of samples. Macro-recall refers to calculating the recall rate for each category and then taking the unweighted average. Precision is the proportion of correctly identified positive classes [33] and is also calculated from the perspectives of micro-precision and macro-precision. The kappa coefficient measures whether the actual classification results of the model are consistent with the prediction results [34, 35]. The Matthews correlation coefficient (MCC) describes the correlation between the real and predicted classification [36, 37]. Hamming loss measures the distance between the real and predicted labels [38], where a value closer to 0 indicates a better prediction result. The Jaccard score is opposite to Hamming loss, as a value closer to 1 indicates a better prediction result. For multi-classification problems, we can also use the macro-roc and micro-roc curves to evaluate the performance of the model. The area enclosed by the ROC curve and the horizontal and vertical coordinates is the AUC value, and the AUC value is closer to 1, the better the classification performance of the model.

The confusion matrix in Table 4 can help us to calculate the classification performance of each model. The following formulas were used to calculate the evaluation indicators used in this paper:

$$Micro-\mathrm{Pr}ecision=\frac{\sum_{i=1}^{n}TP_{i}/n}{\sum_{i=1}^{n}TP_{i}/n+\sum_{i=1}^{n}FP_{i}/n}$$ (4)

$$Macro-\mathrm{Pr}ecision=\frac{\sum_{i=1}^{n}\left[TP_{i}/(TP_{i}+FP_{i})\right]}{n}$$ (5)

$$Micro-Recall=\frac{\sum_{i=1}^{n}TP_{i}/n}{\sum_{i=1}^{n}TP_{i}/n+\sum_{i=1}^{n}FN_{i}/n}$$ (6)

Macro−Recall=∑i=1n[TPi/(TPi+FNi)]n (7)

$$K\mathrm{appa}=\frac{\mathrm{Pr}(a)-\mathrm{Pr}(e)}{1-\mathrm{Pr}(e)}$$ (8)

$$MCC=\frac{C\times S-\sum_{l}^{L}p_{l}\times t_{l}}{\sqrt{\left(S^{2}-\sum_{l}^{L}p_{l}^{2})\times (S^{2}-\sum_{l}^{L}t_{l}^{2}\right)}}$$ (9)

$$Ham\min g-loss=\sum_{i=1}^{n_{labels}}1(y_{i}\neq {\hat{y}}_{i})/n_{labels}$$ (10)

$$Jaccard-score=\frac{\sum_{l\in L}\left[(y_{i}\cap {\hat{y}}_{i})/(y_{i}+{\hat{y}}_{i}-y_{i}\cap {\hat{y}}_{i}\right)]}{L}$$ (11)

where *n_labels_* and L are the numbers of categories, S is the total number of samples, C is the total number of correctly predicted samples, *t_i_* is the number of samples in category i, *p_i_* is the number of samples predicted as category i, *y_i_* is the true value, and ${\hat{y}}_{i}$ is the predicted value.

**Table 4:** Classification confusion matrix.

		actual value	
		1 0	
predicted value	1	TP	FP
	0	FN	TN

## Results and discussion

In this section, we compared the multi-classification performances of seven machine learning models with different evaluation indicators. We derived important impact indicators on the classification of national physical fitness test results based on feature importance scores.

### Classification results

We applied seven classification methods to classify fitness test results. By comparing different evaluation indicators, the optimal machine learning algorithm for the classification of physical fitness test grades was obtained.

Table 5 compares the seven machine learning classification algorithms. MCC and the kappa coefficient take values from –1 to 1, and a value closer to 1 indicates better classification results. Hamming loss is between 0 and 1, and a value closer to 0 is better. Other evaluation indicators range from 0 to 1, where a value closer to 1 indicates a better result. From the table, we can see that, regardless of the evaluation index, the MLP results were the best. In the MCC evaluation index, the classification result of MLP was the best, at 0.637, and the classification result of LR was the worst, at 0.459. For other evaluation indicators, MLP classification results were the best, and LR classification results were the worst.

**Table 5:** Comparison of classification results.

Classifier Algorithm	Micro-precision	Macro-precision	Micro-recall	Macro-recall	MCC	Kappa	Jaccard-score	Hamming-loss
LR	**0.594**	**0.577**	**0.594**	**0.599**	0.463	**0.458**	**0.423**	**0.406**
ABM1	0.636	0.642	0.636	0.637	0.514	0.513	0.478	0.364
MLP	**0.728**	**0.744**	**0.728**	**0.729**	**0.637**	**0.635**	**0.584**	**0.272**
KNN	0.617	0.648	0.617	0.612	0.487	0.484	0.456	0.383
DT	0.693	0.708	0.693	0.696	0.590	0.588	0.544	0.307
RF	0.717	0.730	0.717	0.722	0.623	0.622	0.572	0.283
SVM	0.597	0.606	0.597	0.600	**0.459**	0.459	0.438	0.403

**Fig 8:**
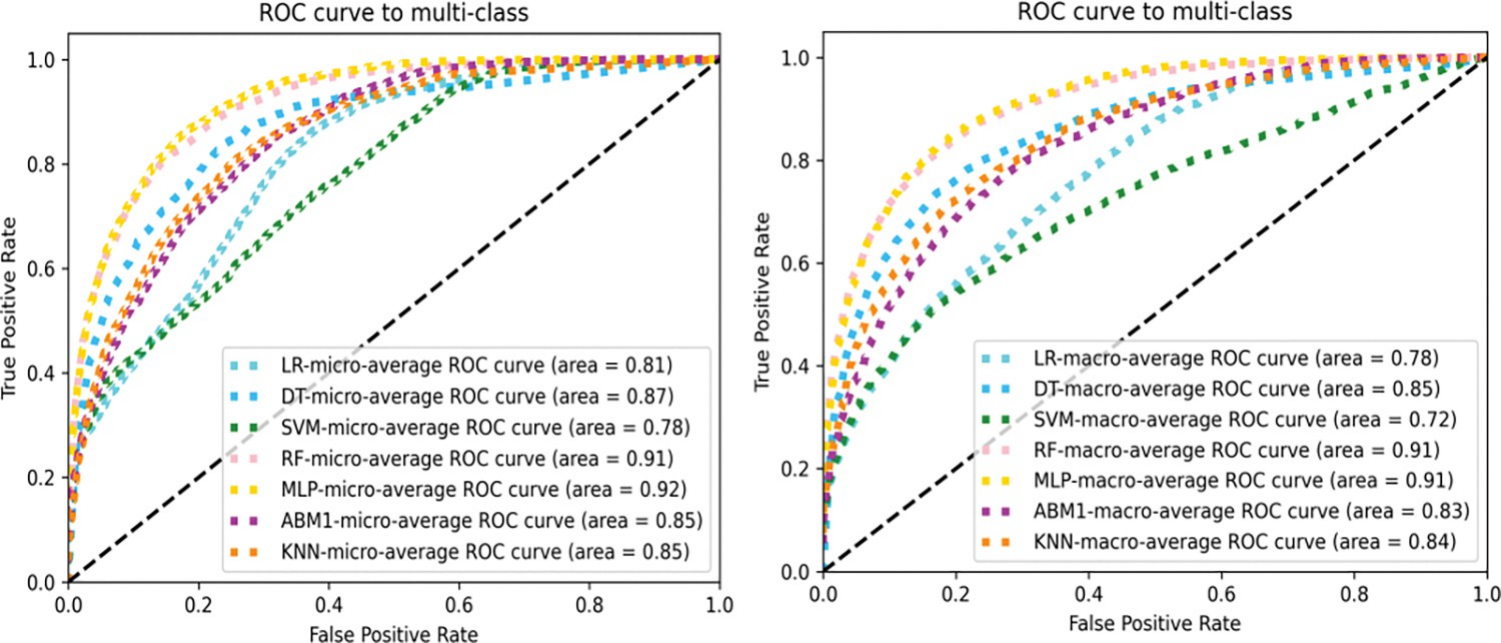
Micro-roc and macro-roc plots.

Fig 8 shows the AUC values corresponding to the ROC curves calculated using "micro" and "macro" under seven machine learning classification algorithms. Under macro-roc, the AUC values of RF and MLP were the same, at 0.91, indicating that they were the best, followed by DT, with an AUC value of 0.85, where the minimum AUC value corresponding to SVM was 0.72. Under micro-roc, the AUC value of MLP was the highest, at 0.92, followed by RF, at 0.91, where the minimum AUC value, corresponding to SVM, was 0.78. For both micro-roc and macro-roc, the best classifier was MLP, and the worst was SVM.

We compared the classification performance of seven machine learning algorithms on nine evaluation indices. From Table 5 and Fig 8, the MLP algorithm was consistently the best. From the MCC, micro-roc, and macro-roc evaluation indicators, it can be concluded that the worst classification algorithm was SVM, and that for other evaluation indicators, the worst was LR.

### Feature importance selection results

Feature importance plays an important role in modeling, and this method reveals the relative importance of each feature. The importance and ranking of each feature are determined according to its coefficient value, and deleting unimportant features or retaining important ones can improve the performance of the model. Because MLP and KNN cannot calculate feature importance or coefficient scores, we use five supervised machine learning algorithms to calculate the feature importance or coefficient score, which are ABM1, LR, RF, DT, and SVM.

**Fig 9:**
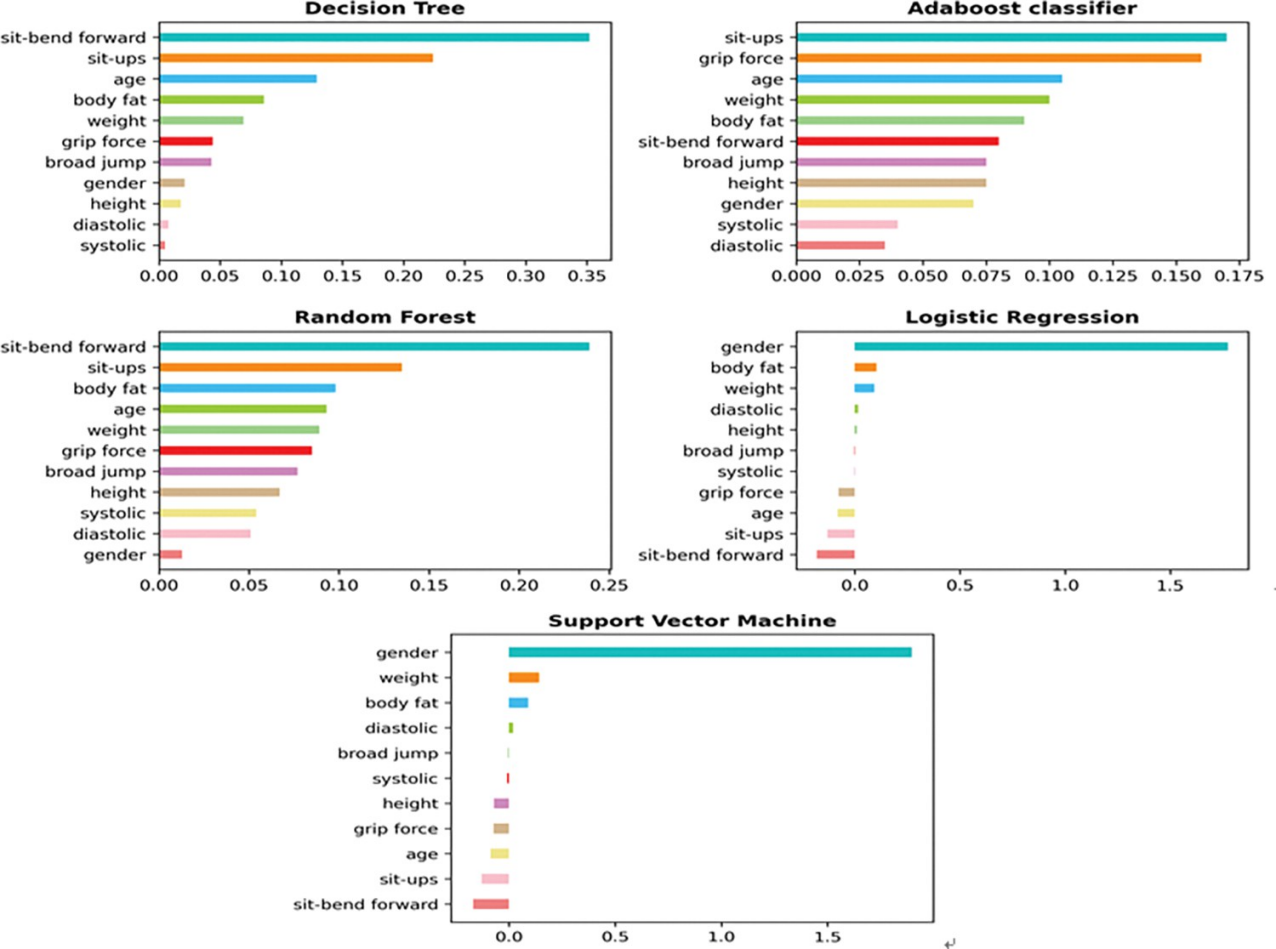
Bar charts of feature importance; from top to bottom, the feature importance decreases.

The feature importance and coefficient score results calculated by the five supervised machine learning algorithms are shown in Table 6. The larger their values, the more important the corresponding features are to the model classification. Fig 9 uses bar graphs to more clearly show the rankings of each feature under different algorithms.

**Table 6:** Feature importance score under different classifiers.

Feature	Tree	ABM1	RF	LR	SVM
Age	0.129	0.105	0.093	−0.081	−0.087
Gender	0.021	0.070	0.013	1.773	1.895
Height	0.018	0.075	0.067	0.010	−0.070
Weight	0.069	0.100	0.089	0.092	0.141
Body fat	0.086	0.090	0.098	0.102	0.090
Diastolic	0.008	0.035	0.051	0.016	0.020
Systolic	0.005	0.040	0.054	−0.003	−0.008
Grip force	0.044	0.160	0.085	−0.077	−0.073
Sit-bend forward	0.352	0.080	0.239	−0.181	−0.170
Sit-ups	0.224	0.170	0.135	−0.131	−0.130
Broad jump	0.043	0.075	0.077	−0.003	−0.004

As we can see from Table 7, under the five machine learning algorithms, the five most important features were selected according to the feature importance and coefficient scores. It can be concluded that DT was similar to RF in selecting the five most important features, although their orders of importance were inconsistent. Of the features selected using DT and ABM1, only one was different, and the same was true for LR and SVM. In addition, the characteristics of weight and body fat rank among the top five for each machine learning algorithm, which are important indicators for the classification of fitness test results. Sit-ups, age, sit-bend forward, and systolic were also important factors that lead to different grades of physical fitness test results.

**Table 7:** Five most important features of five different machine algorithms.

	DT	ABM1	RF	LR	SVM
1st	Sit-bend forward	Sit-ups	Sit-bend forward	Gender	Gender
2nd	Sit-ups	Grip force	Sit-ups	**Body fat**	**Weight**
3rd	Age	Age	**Body fat**	**Weight**	**Body fat**
4th	**Body fat**	**Weight**	Age	Diastolic	Diastolic
5th	**Weight**	**Body fat**	**Weight**	Height	Broad jump

From the perspective of reducing the cost of health detection, combined with the accuracy of machine learning technology in classification, we used the LR, ABM1,MLP, KNN, DT, RF,and SVM classifiers to classify physical fitness test results into grades of excellent, good, pass, and fail. In summary, the novelty of this work is mainly reflected in the following three aspects:

Statistical analysis and machine learning methods were used for the first time to classify national physical fitness test results. Our investigation revealed that the classical machine learning algorithm has high accuracy and excellent potential utility for the classification of physical fitness level. Using ten evaluation indicators to compare seven classification models, we concluded that MLP was the best multi-classification model.

The selected measurement indicators come from a social and physical fitness test survey aimed at assessing national health. These indicators are both scientific and practical. Through the above statistical analysis, we concluded that the variables age, gender, weight, height, body fat, diastolic, systolic, grip force, sit-bend forward, sit-ups, and broad jump had significant effects on fitness test results.

Except for MLP and KNN, all applied classifier models estimate the importance score of each measurement index and rank them according to the feature importance score. It was found that the variables sit-ups, age, sit-bend forward, systolic, weight, and body fat were important impact indicators of the grade classification of physical fitness test results. Moreover, the feature importance of the variables weight and body fat were among the top five in each classification model.

## Conclusion

Scientifically guiding people to participate in physical exercise is crucial to improving physical health. This paper evaluated an individual’s physical health status from the perspective of non-medical, low-cost physical fitness testing. We build a reasonable health indicator system based on national physical examination data, break group restrictions, study national groups, and hope to use machine learning models to provide useful health suggestions for citizens to measure their physical status. The research showed that the MLP has the best classification effect among the seven machine learning models. Among the selected features, it was found that weight, body fat, sit-bend forward, and systolic were important influencing indicators for the classification of physical fitness test results. It is hoped that individuals use the multi-classification model in this paper to understand their physical fitness level through physical fitness test data and conduct appropriate physical exercise based on the measurement indicators. Physique testing does not diagnose diseases but helps us to understand our physical condition and health level, formulate an exercise plan, and enhance physical fitness and health while providing a scientific basis for organizing people to participate in sports.

There are still certain limitations in current research. Although the accuracy of the classification model has reached satisfactory results, a single machine learning model was used to classify national physical fitness test levels, and the classification efficiency needs to be improved to save time and cost. For future works, model fusion technology is an essential step in a multi-classifier system. We intend to improve the performance of a single model by fusing multiple machine learning models to make the classification of national physical fitness test results more accurate and better reduce computing costs. In addition, regarding the construction of physical measurement indicators, although we have established a reasonable indicator system that can help people exercise scientifically, how to formulate practical exercise plans based on indicator test data still needs to be solved.

## Supporting information

S1 Table Raw data. (CSV)

S2 Table Data after outlier processing. (CSV)
